# Apatinib with doxorubicin and ifosfamide as neoadjuvant therapy for high-risk soft tissue sarcomas: a retrospective cohort study

**DOI:** 10.1007/s10637-021-01139-w

**Published:** 2021-06-22

**Authors:** Zhichao Tian, Jiaqiang Wang, Jinpo Yang, Peng Zhang, Xin Wang, Fan Zhang, Po Li, Weitao Yao

**Affiliations:** 1 Department of Bone and Soft Tissue, the Affiliated Cancer Hospital of Zhengzhou University and Henan Cancer Hospital, Henan Province, Zhengzhou, 450008 China; 2 Department of Medical Oncology, the Affiliated Cancer Hospital of Zhengzhou University and Henan Cancer Hospital, Henan Province, Zhengzhou, 450008 China

**Keywords:** Apatinib, Doxorubicin, Ifosfamide, Sarcoma, Neoadjuvant therapy

## Abstract

***Background*:**

There is a need to establish an effective neoadjuvant therapy for soft tissue sarcomas (STSs). We previously showed that apatinib, administered in combination with doxorubicin-based chemotherapy, improves the efficacy of treatment. This study aimed to clarify the effectiveness and safety of apatinib combined with doxorubicin and ifosfamide (AI) neoadjuvant chemotherapy for STSs.

***Methods*:**

This retrospective study included patients with STS who received neoadjuvant therapy and surgery between January 2016 and January 2019. The patients were divided into two treatment groups: AI + apatinib group and AI group (doxorubicin + ifosfamide).

***Results*:**

The study included 74 patients (AI + apatinib: 26, AI: 48) with STS. There were significant between-group differences in objective response rates (53.85% vs. 29.17%, p = 0.047) and the average change in target lesion size from baseline (-40.46 ± 40.30 vs. -16.31 ± 34.32, p = 0.008). The R0 rate (84.62% vs. 68.75%; p = 0.170) and 2-year disease-free survival (73.08% vs. 62.50%, p = 0.343) were similar across groups. Finally, the rates of neoadjuvant therapy-related adverse effects and postoperative complications were similar in both groups (p > 0.05).

***Conclusion*:**

Apatinib plus doxorubicin and ifosfamide regimen is safe and effective as neoadjuvant therapy for patients with STS. However, the significantly improved preoperative ORR observed after neoadjuvant therapy did not translate into a significantly improved R0 rate and 2-year DFS. Prospective, well-powered studies are warranted to determine the long-term efficacy and optimal application of these protocols.

## Background

There are over 70 subtypes of soft tissue sarcomas (STSs) [1]. Although rare, STS accounts for approximately 40,000 new diagnoses in China each year [2]. The standard treatment for localized STS is surgical resection [3]. Despite achieving optimal local control, over 50% of patients with localized STS succumb to metastatic disease [4]. The first-line treatment for advanced (locally unresectable or metastatic) STS is chemotherapy with doxorubicin [3]. The overall response rate (ORR) to this treatment for advanced STS is approximately 20% [5], and the 5-year survival rate among patients with advanced STS treated with a combination regimen is < 10% [6]. These findings suggest the need for an approach that may help reduce the rates of recurrence and metastasis in patients with early- and mid-stage STS. Neoadjuvant chemotherapy (preoperative adjuvant chemotherapy) is a candidate approach in this context [7].

Despite this need, the efficacy of neoadjuvant chemotherapy for STS remains controversial as evidence from clinical trials has failed to convincingly demonstrate the effectiveness of neoadjuvant chemotherapy for STS [8–10]. Due to the ongoing debate over the efficacy of neoadjuvant chemotherapy, the STS research community worldwide is examining ways to improve the efficacy of neoadjuvant therapy [10–13]. This improvement can be achieved by using more sensitive treatment methods or implementing individualized therapy based on sarcoma subtypes. Determining an effective neoadjuvant therapy remains an ongoing research priority.

Apatinib is a multi-target tyrosine kinase inhibitor (TKI), marketed in China, that effectively treats some types of STS [14, 15]. As a leading sarcoma treatment center in central China, we have treated many patients with STS with apatinib [14, 16]. In fact, we previously showed that apatinib combined with doxorubicin was more effective than doxorubicin alone in reducing the size of target lesions in patients with STS [17]. This finding suggests that the use of apatinib combined with doxorubicin-based chemotherapy may improve the efficacy of neoadjuvant therapy. Based on this evidence, we treated some STS patients with apatinib combined with doxorubicin and ifosfamide (AI) neoadjuvant chemotherapy over the past few years. In this study, we retrospectively examined these patients’ clinical data to clarify the effectiveness and safety of apatinib combined with neoadjuvant chemotherapy for treating STS. The present findings may provide a reference for clinical treatment decision-making and future clinical trial design.

## Material and methods

### Patients and eligibility criteria

This retrospective study included patients with STS treated at the Affiliated Cancer Hospital of Zhengzhou University between January 2016 and January 2019. Patients were included in the present study if they: 1) had pathologically confirmed STS, 2) were identified as high-risk patients without evidence of distant metastasis [18], 3) received two cycles of AI or AI + apatinib neoadjuvant therapy, 4) underwent resection of the primary lesion, and 5) had complete follow-up data.

This study was approved by the Ethics Committee of the Affiliated Cancer Hospital of Zhengzhou University. Included patients provided written informed consent for their participation. The study complied with the Declaration of Helsinki guidelines and any other relevant reporting or ethical guidelines.

### Treatment protocol

Patients were divided into AI + apatinib and AI groups based on the type of neoadjuvant therapy they received. In the AI + apatinib group, patients were administered 37.5 mg/m^2^ doxorubicin per day in the form of a short infusion on days 1 and 2; and 2 g/m^2^ of ifosfamide day in the form of an intravenous bolus on days 1–3. The treatment procedure was repeated on day 21. Simultaneously, patients in parallel received 500 mg apatinib once daily, starting on day 1. Apatinib was discontinued on day 35.

In the AI group, patients were administered 37.5 mg/m^2^ of doxorubicin per day in the form of a short infusion on days 1 and 2; and 2 g/m^2^ ifosfamide per day of an intravenous bolus on days 1–3. The treatment procedure was repeated on day 21.

Patients were assessed for signs of toxicity, according to the National Cancer Institute Common Terminology Criteria for Adverse Events version 4.0. In cases of severe toxicity, treatment with apatinib and doxorubicin was delayed until patient recovery, for a maximum of 14 days.

### Surgical resection

Extensive resection of the primary lesion was performed on days 35–45. Patients were confirmed to be free of grade 3–4 adverse events (AEs) at the time of surgery. All surgeries were performed by an experienced STS surgical team. Each surgery aimed to achieve macroscopically complete resection of the tumor mass based on preoperative assessment and intraoperative findings. All operations were routine and non-minimally invasive. No patients received further apatinib or chemotherapy after surgery. All patients received adjuvant radiotherapy after surgery.

### Evaluation

The effectiveness of neoadjuvant therapy was evaluated preoperatively with enhanced magnetic resonance imaging and computed tomography scans, according to the Response Evaluation Criteria in Solid Tumors (version 1.1). Between-group differences in the ORR, target lesion diameter changes from baseline, R0 rate, and 2-year disease-free survival (DFS) were assessed. DFS was defined as the time from surgical resection to signs of recurrence or metastasis or disease-related death, whichever occurred first. The rates of neoadjuvant therapy-related and surgical resection-related AEs were compared between the groups. Surgical resection-related AEs were graded by the Clavien-Dindo grading system.

### Statistical analysis

All statistical analyses were performed using SPSS 21.0 software for Windows. Data are presented as medians (range) or counts (percentage). The Wilcoxon rank-sum test with continuity correction was used to analyze continuous variables. Fisher’s exact test was used for the analysis of categorical variables. All statistical analyses were two-sided, and p-values < 0.05 were considered indicative of a statistically significant difference. This was a descriptive analysis.

## Results

### Patients’ characteristics

A total of 74 patients with STS met the eligibility criteria for this study and subsequently assigned to the AI + apatinib (n = 26) and AI (n = 48) groups. The patients’ baseline characteristics were similar between groups and are presented in Table 1. Both groups featured more females than males. The median ages of patients in the AI + apatinib and AI groups were 42.04 ± 14.84 and 44.52 ± 13.34 years. The group members’ Eastern Cooperative Oncology Group Performance Status scores ranged from 0–1. The primary lesions were most commonly located in the extremities, followed by the trunk and the head and neck. The distribution of histological subtypes in the AI + apatinib group was as follows: undifferentiated sarcoma (n = 7), synovial sarcoma (n = 6), leiomyosarcoma (n = 4), angiosarcoma (n = 4), fibrosarcoma (n = 3), rhabdomyosarcoma (n = 1), and malignant peripheral nerve sheath tumor (MPNST) (n = 1). The distribution of histological subtypes in the AI group was as follows: undifferentiated sarcoma (n = 9), synovial sarcoma (n = 12), leiomyosarcoma (n = 11), angiosarcoma (n = 3), fibrosarcoma (n = 4), rhabdomyosarcoma (n = 5), MPNST (n = 2), and liposarcoma (n = 2). The mean diameters of primary lesions in the AI + apatinib and AI groups were 10.13 ± 5.21 and 9.89 ± 4.36 cm, respectively. Baseline characteristics were similar between the groups (Table 1).

**Table 1:** Patient characteristics by treatment group

Characteristics	AI + apatinib group (n = 26)	AI group (n = 48)	*P—*value
Sex			0.807
Male	11 (42.31%)	23 (47.92%)	
Female	15 (57.69%)	25 (52.08%)	
Median age (years)	42.04 ± 14.84	44.52 ± 13.34	0.465
ECOG PS			1.000
0	21 (80.77%)	37 (77.08%)	
1	5 (19.23%)	11 (22.92%)	
2	0	0	
Primary tumor site			0.835
Extremities	16 (61.54%)	26 (54.17%)	
Trunk	7 (30.77%)	15 (31.25%)	
Head and neck	3 (11.54%)	7 (14.58%)	
Histological types			0.768
Undifferentiated sarcoma	7 (30.77%)	9 (18.75%)	
Synovial sarcoma	6 (23.08%)	12 (25.00%)	
Leiomyosarcoma	4 (15.38%)	11 (22.92%)	
Angiosarcoma	4 (15.38%)	3 (6.25%)	
Fibrosarcoma	3 (11.54%)	4 (8.33%)	
Rhabdomyosarcoma	1 (3.85%)	5 (10.42%)	
MPNST	1 (3.85%)	2 (4.17%)	
Liposarcoma	0	2 (4.17%)	
Mean tumor size (cm)	10.13 ± 5.21	9.89 ± 4.36	0.910

Data are presented as counts (percentages) or means ± standard deviations

*AI* neoadjuvant chemotherapy with doxorubicin and ifosfamide, *ECOG PS* Eastern Cooperative Oncology Group performance status, *MPNST* malignant peripheral nerve sheath tumor

### Effectiveness of the treatment

**Fig. 1:**
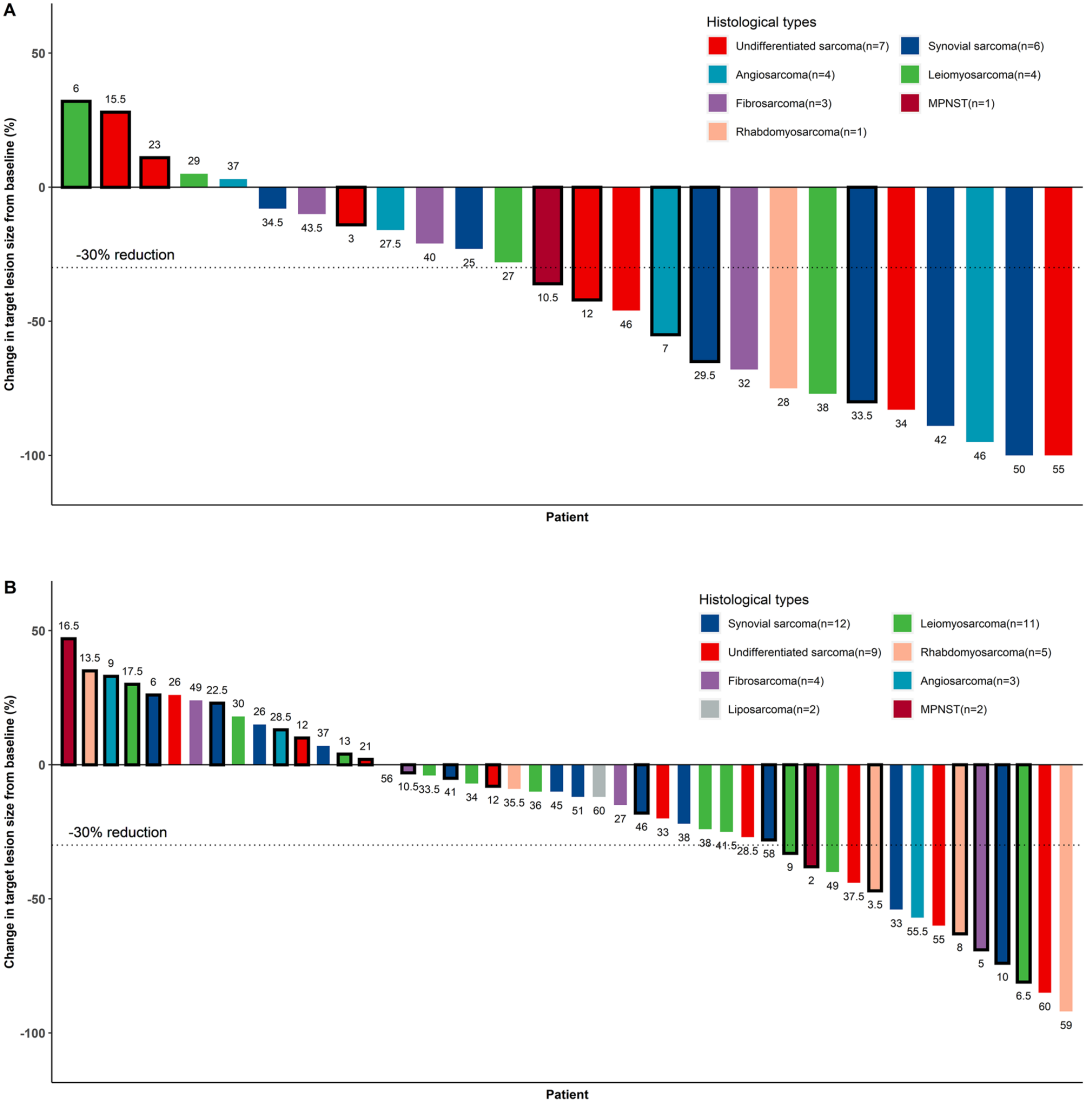
Maximum percentage diameter changes from baseline in target lesion for AI + apatinib group (**A**) and AI group (**B**). Treatment effectiveness was evaluated with the Response Evaluation Criteria in Solid Tumors version 1.1 (RECIST). Black circles represent patients that experienced recurrence or metastasis. The number at the top of the cylinder represents estimated disease-free survival for each patient. MPNST, malignant peripheral nerve sheath tumor

Neoadjuvant therapy effectiveness was evaluated preoperatively, after administration of neoadjuvant therapy. In the AI + apatinib group, one patient with undifferentiated sarcoma and another with synovial sarcoma achieved complete response (CR) (Fig. 1). In contrast, no patient in the AI group achieved CR. There were significant between-group differences in ORR (53.85% vs. 29.17%, p = 0.047; Table 2) and the average change in target lesion size from baseline (- 40.46 ± 40.30 vs. -16.31 ± 34.32, p = 0.008; Table 2 and Fig. 1).

**Table 2:** Effectiveness of the treatment

Characteristics	AI + apatinib group (n = 26)	AI group (n = 48)	*P-* value
ORR	53.85% (14/26)	29.17% (14/48)	0.047
Diameter changes from baseline in target lesion (mm)	- 40.46 ± 40.30	-16.31 ± 34.32	0.008
R0 rate	84.62% (22/26)	68.75% (33/48)	0.170
DFS rate (2-year)	73.10%	62.50%	0.343

Data are presented as numbers (percentages) or means ± standard deviations

*AI* neoadjuvant chemotherapy with doxorubicin and ifosfamide, *ORR* objective response rate, *DFS* disease-free survival

**Fig. 2:**
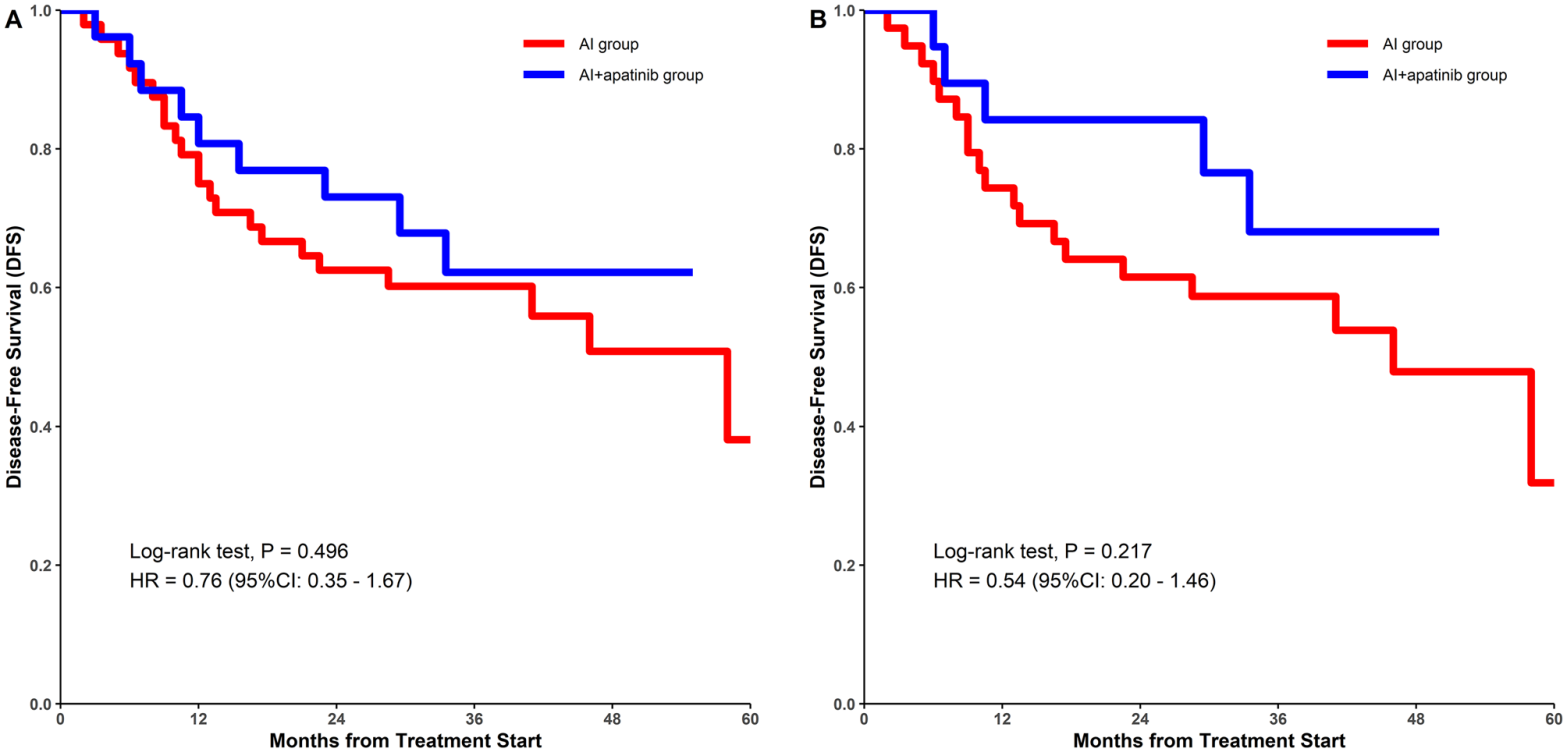
Kaplan–Meier estimates of disease-free survival for both treatment groups of all the patients (**A**) or patients Excluded undifferentiated sarcoma (**B**)

Postoperative effectiveness evaluation included R0 excision rate and 2-year DFS. There was no significant difference in R0 rate (84.62% vs. 68.75%; p = 0.170; Table 3) or the 2-year DFS (73.08% vs. 62.50%, p = 0.343; Table 2 and Fig. 2).

**Table 3:** Effectiveness of the treatment in no undifferentiated sarcoma patients

Characteristics	AI + apatinib group (Excluded undifferentiated sarcoma, n = 19)	AI group (Excluded undifferentiated sarcoma, n = 39)	*P-* value
ORR	52.63% (10/19)	28.21% (11/39)	0.086
Diameter changes from baseline in target lesion (%)	-42.42 ± 38.81	-14.79 ± 34.36	0.008
R0 rate	89.47% (17/19)	64.10% (25/39)	0.061
DFS rate (2-year)	84.20%	61.51%	0.047

Data are presented as numbers (percentages) or means ± standard deviations

*AI* neoadjuvant chemotherapy with doxorubicin and ifosfamide, *ORR* objective response rate, *DFS* disease-free survival

### Ancillary analysis

To investigate the effect of sarcoma histological subtypes on neoadjuvant therapy outcomes, we evaluated treatment outcomes after excluding patients with undifferentiated sarcoma. As shown in Table 3 and Fig. 2, we found a significant between-group difference in 2-year DFS (84.20% vs. 61.51%, P = 0.047).

### Toxicity evaluation

The major neoadjuvant therapy-related AEs observed in the groups are presented in Table 4. Neoadjuvant therapy-related AEs were more common in the AI + apatinib group than in the AI group; however, this difference did not rise to the level of statistical significance (p > 0.05, Table 4). Most patients experienced grade 1 or 2 AEs, and a few patients experienced grade 3 or 4 AEs. No drug-related deaths occurred.

**Table 4:** Neoadjuvant therapy-related adverse effects per treatment groups

Characteristics	AI + apatinib group (n = 26)		AI group (n = 48)		*p*-value	
	All grades	Grade > 2	All grades	Grade > 2	All grades	Grade > 2
Leukopenia	21 (80.77%)	11 (42.31%)	36 (75.00%)	14 (29.17%)	0.773	0.307
Fatigue	20 (76.92%)	4 (15.38%)	30 (62.50%)	3 (6.25%)	0.299	0.232
Anemia	19 (73.08%)	8 (30.77%)	32 (66.67%)	11 (22.92%)	0.610	0.579
Thrombocytopenia	19 (73.08%)	7 (26.92%)	33 (68.75%)	10 (20.83%)	0.793	0.574
Oral mucositis	18 (69.23%)	5 (19.23%)	25 (52.08%)	2 (4.17%)	0.218	0.089
Vomiting	16 (61.54%)	8 (30.77%)	29 (60.42%)	9 (18.75%)	1.000	0.260
Anorexia	18 (69.23%)	3 (11.54%)	23 (47.92%)	4 (8.33%)	0.092	0.691
Transaminase increase	12 (46.15%)	3 (11.54%)	17 (35.42%)	3 (6.25%)	0.456	0.659

Data are presented as counts (percentages)

*AI* neoadjuvant chemotherapy with doxorubicin and Ifosfamide

Postoperative complications per treatment group are presented in Table 5. Grade IV (Clavien-Dindo grading) complications—including cardiac failure and deep venous thrombosis—occurred once in the AI + apatinib and AI groups, respectively. No perioperative deaths occurred. There was no statistically significant between-group difference in the incidence of postoperative complications (P > 0.05, Table 5).

**Table 5:** Postoperative complications per treatment group

Complication	AI + apatinib group (n = 26)	AI group (n = 48)	*p*—value
Clavien-Dindo grading			0.538
Grade I	1	2	
Grade II	4	2	
Grade III	2	6	
Grade IV	1	1	
Grade V	0	0	
Wound infection	2 (7.69%)	2 (4.17%)	0.609
Pulmonary infection	3 (11.54%)	2 (4.17%)	0.337
Hemorrhage	1 (3.85%)	3 (6.25%)	1.000
Superficial wound dehiscence	1 (3.85%)	2 (4.17%)	1.000
Cardiac/respiratory failure	1 (3.85%)	0	0.351
Cerebral infarction	0	1 (2.08%)	1.000
Deep venous thrombosis	0	1 (2.08%)	1.000
Readmission	1 (3.85%)	1 (2.08%)	1.000
Reoperation	1 (3.85%)	0	0.351
Death	0	0	1.000

Data are presented as counts (percentages)

*AI* neoadjuvant chemotherapy with doxorubicin and Ifosfamide

## Discussion

**Fig. 3:**
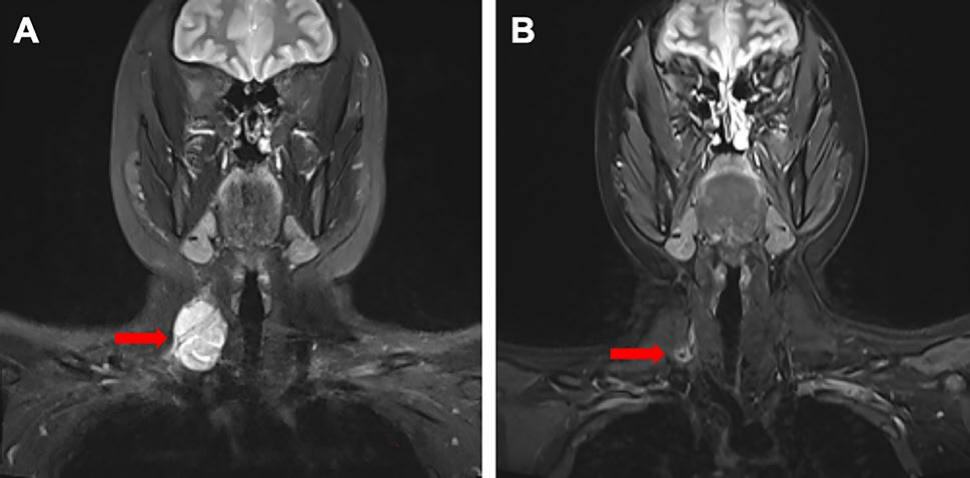
Magnetic resonance imaging scan of a synovial sarcoma patient before neoadjuvant therapy (**A**) and before resection (**B**). The red arrow indicates the primary lesion

Previous studies have demonstrated that combinations of multi-target TKIs and cytotoxic chemotherapy can overcome chemoresistance [15, 19]. Apatinib may act as an effective chemotherapy sensitizer for reducing doxorubicin-induced chemoresistance [20]. The present study findings support this conclusion. This study’s ORR was higher than the previous study [17]. This increase was likely due to the addition of ifosfamide to the chemotherapy regimen. The aim of neoadjuvant therapy is to reduce the diameter of the target lesion, thus simplifying surgery (Fig. 3). Nevertheless, neoadjuvant therapy may increase the risk of disease progression, rendering surgery impossible. This suggests that an intensive neoadjuvant regimen may be required to concurrently minimize the risk of disease progression and reduce the target lesion size. Doxorubicin plus ifosfamide can reduce the diameter of target lesions more than doxorubicin alone [21]. Based on these findings, we used the AI regimen as neoadjuvant chemotherapy.

Compared with the ORR associated with AI, that associated with AI + apatinib was significantly improved in patients with STS; similar findings were observed for target lesion shrinkage. Moreover, the rates of R0 and 2-year DFS in the AI + apatinib group were higher than in the comparison group. However, the differences in R0 rate and 2-year DFS were not statistically significant. The error caused by the small sample size may be one reason for the non-significant between group differences in R0 rate and 2-year DFS observed in this study. In addition, R0 and 2-year DFS were significantly different between the two groups after excluding patients with undifferentiated sarcoma. This suggests that the difference in histological subtypes between the two groups is another reason why the significant ORR in the preoperative evaluation in this study did not convert to a significant R0 and DFS postoperatively. Some patients with other histological subtypes in this study responded well preoperatively but developed recurrence/metastasis shortly after surgery (Fig. 1). This suggests that, for some sarcoma subtypes, higher preoperative ORR does not translate into prolonged postoperative DFS.

Aside from sarcoma subtypes, other factors contribute to preoperative neoadjuvant therapy failure. One such error involves image evaluation. For example, although a patient was recognized as having CR based on imaging findings in evaluating neoadjuvant therapy, postoperative pathology tests confirmed the presence of residual tumor (Fig. 3). There are many other methods to evaluate the perioperative efficacy of neoadjuvant therapy—including imaging, pathological necrosis rate, R0 assessment, etc. However, it is unclear which method most accurately predicts DFS [4, 22, 23]. Well-powered prospective studies are required to answer these questions. In conclusion, although better perioperative outcomes of neoadjuvant therapy do not always translate into better postoperative DFS, better postoperative DFS requires better perioperative outcomes. We can conclude based on these findings that AI + apatinib achieves superior perioperative effectiveness compared to AI alone.

Neoadjuvant therapy safety is important. Complications associated with neoadjuvant therapy can delay surgery and prolong the overall treatment time. In the present study, the incidence of neoadjuvant treatment-related AEs was similar between groups, as was incidence of postoperative complications. However, we did not rigorously screen patients ahead of enrollment. Patients in a better overall condition were inadvertently more likely to receive combination therapy than their counterparts. This should be considered when reviewing this study’s safety assessment. Nevertheless, the present safety-related findings are consistent with previous studies that used TKIs in combination with chemotherapy for STS [24, 25]. These findings suggest the safety of AI + apatinib as neoadjuvant therapy for STS.

This study had some limitations. This was a retrospective study with small sample size, resulting in low-level evidence. These limitations notwithstanding, our findings suggest that AI + apatinib may be a promising neoadjuvant therapy for STS. It remains unclear whether the perioperative evaluation of neoadjuvant therapy efficacy can support patient prognostication. Different histological subtypes may have different outcomes. Long-term, prospective studies are required to evaluate these considerations.

## Conclusions

In conclusion, the apatinib plus doxorubicin and ifosfamide regimen is safe and effective as neoadjuvant therapy for STS. However, the significantly improved preoperative ORR observed after neoadjuvant therapy did not translate into a significantly improved R0 rate and 2-year DFS. Prospective, well-powered studies are warranted to determine the long-term efficacy and optimal application of these protocols.

## Data Availability

The datasets used and/or analyzed during the current study are available from the corresponding author on reasonable request.
